# The Effects of Beta-Glucan Rich Oat Bread on Serum Nitric Oxide and Vascular Endothelial Function in Patients with Hypercholesterolemia

**DOI:** 10.1155/2014/481904

**Published:** 2014-06-12

**Authors:** Faezeh Tabesh, Hamid Sanei, Mansour Jahangiri, Amir Momenizadeh, Elham Tabesh, Kiana Pourmohammadi, Masoumeh Sadeghi

**Affiliations:** ^1^Medical Students' Research Center, Isfahan University of Medical Sciences, P.O. Box 81465-1148, Isfahan, Iran; ^2^Cardiac Rehabilitation Research Center, Isfahan Cardiovascular Research Institute, Isfahan University of Medical Sciences, Khorram Avenue, P.O. Box 81465-1148, Isfahan, Iran; ^3^Department of Cardiology, Isfahan University of Medical Sciences, P.O. Box 81465-1148, Isfahan, Iran; ^4^Department of Internal Medicine, Isfahan University of Medical Sciences, P.O. Box 81465-1148, Isfahan, Iran; ^5^Department of Nutrition, Mashhad University of Medical Sciences, Mashhad, Iran

## Abstract

*Introduction*. Oats are high in soluble fibers and effective in reducing the risk of cardiovascular diseases (CVD). We assessed the effects of beta-glucan from oat bran on serum nitric oxide (NO) endothelial function in patients with hypercholesterolemia. *Method*. Sixty hypercholesterolemic patients were randomly divided to receive an experimental bread rich in beta-glucan from oat bran (intervention) or bread rich in wheat fiber (control) for four weeks. All subjects had the same diet for two-week baseline period and hypocaloric diet for four weeks of intervention. Serum NO concentration and flow-mediated dilation (FMD) were determined before and after the experiment. *Results*. Mean age of the participants was 51.1 ± 9.3 years and 65% (*n* = 39) were female. After intervention, serum NO concentration increased by 50.2 ± 19.8 *μ*mol/lit in the intervention group (*P* = 0.017), but no change was observed in the control group (17.5 ± 27.5 *μ*mol/lit; *P* = 0.530). No change of FMD was observed in the intervention (0.48 ± 0.78%; *P* = 0.546) or in the control group (0.59 ± 0.92%; *P* = 0.533). *Conclusion*. Consumption of oat bread for four weeks increases serum NO concentration but has no effect on FMD. Further studies are warranted in this regard.

## 1. Introduction

Cardiovascular diseases (CVD) are among the most common causes of death and disability [1]. Old age, obesity, physical inactivity, high blood pressure, cigarette smoking, alcohol intake, and dyslipidemia are among the risk factors for CVD and modifiable factors must be considered for the prevention of CVD [2, 3]. Dyslipidemia, one of the major risk factors, is characterized by elevated levels of low-density lipoprotein (LDL), high concentrations of serum total cholesterol, and reduced high-density lipoprotein (HDL). Both genetic and environmental factors affect the plasma lipid profile [4].

It is well established that diet and nutrition have a direct effect on pathological conditions like obesity, hypertension, and CVD. Diets which are rich in fruits, vegetables, bread, cereals, potatoes, beans, nuts, seeds, olive oil, dairy products, and fish are associated with lower risk of CVD [5]. Evidence suggests that increased consumption of dietary fibers, such as beta-glucan, can effectively lower the risk of CVD through targeting the risk factors such as hypercholesterolemia. It has been reported that 1% reduction in serum LDL level is associated with 1% to 2% decrease of CVD occurrence [6–8].

Oats are high in soluble fibers and studies have shown that they can reduce the CVD risk. Cholesterol lowering effect of oats is thought to be because of soluble fiber beta-glucan as an active component [8]. While bread made of wheat is the main type of bread that is consumed in Iran, bran is usually separated from wheat and, therefore, Iranian bread lacks fiber, vitamins, and minerals. Considering that oats are high in soluble fibers and regarding their effects on reducing the CVD risk, we assessed the effects of bread rich in beta-glucan from oat bran on serum nitric oxide (NO) concentration and flow-mediated dilation (FMD) as indicators of endothelial function in Iranian patients with hypercholesterolemia. We hypothesized that beta-glucan rich oat bread is effective in improving the endothelial function in hypercholesterolemic patients.

## 2. Methods

### 2.1. Study Population

This was a randomized controlled clinical trial conducted on a sample of patients with hypercholesterolemia who were referred to the Isfahan Healthy Heart Program [9]. Inclusion criteria were (a) age between 18 to 65 years, (b) hypercholesterolemia as defined by total cholesterol of between 200 and 300 mg/dL [10], (c) serum LDL up to 190 mg/dL and TG less than 300 mg/dL, and (d) no history of the followings; secondary dyslipidemia, diabetes mellitus, hypothyroidism, renal failure, anemia, cholestasis, cancer, history of taking antihypertensive drugs, consumption of alcohol, smoking, professional exercise, eating disorders, and weight changes (losing or gaining more than three-kilogram weight during the three months prior to the study). Sample size was calculated as 30 patients in each group based on *α* = 5%, *β* = 0.2, and SD = 6. The study was approved by the Ethical Committee of the Isfahan University of Medical Sciences and registered at the Iranian Registry of Clinical Trials (IRCT201312073733N3). Informed consent was obtained from all participants.

### 2.2. Intervention

Using a table of random numbers, participants were randomly divided into two groups of intervention and control. For two weeks, all the subjects had the same diet of usual food including wheat bread in order to maintain their constant body weight. After two weeks, all subjects were provided with a hypocaloric diet in the calorie range of 1500–2000 for 4 weeks. Energy intake for each individual was calculated based on maintenance energy needs minus 500–700 kcal/d depending on their body mass index (BMI). In these four weeks, in addition to the hypocaloric diet, the intervention group received an experimental bread rich in beta-glucan from oat bran (at least five servings (150 gr) per day based on their needs, each serving of which had 6 gram beta-glucan). The control group received hypocaloric diet with a bread rich in wheat fiber (at least five servings (150 gr) per day based on their needs, with no beta-glucan). New batches of both experimental and control bread were prepared by a local bakery and then delivered at home to each subject once a week based on their request. In addition, the participants were instructed to keep a detailed 3-day diet record every week that would be reviewed by the dietitian in the next visit during the 6-week protocol. Both groups were asked to continue their routine levels of physical activity and not to consume fiber supplements, weight loss drugs, herbal medicines, or laxatives. During the 6-week course of the intervention, the participants attended weekly visits with an internist and a nutritionist (who evaluated their daily intake of food items).

### 2.3. Assessments

Demographic characteristics of the participants including age and sex were recorded. All participants were examined for height, weight, pulse rate, and systolic and diastolic blood pressure. All measurements were performed with one particular tool. A Seca scale was used for measuring weight. Blood pressures were taken after 20 minutes of rest and in sitting position. Body mass index was calculated as weight divided by height squared. Hip and waist circumference were also measured.

### 2.4. Serum NO Concentration

To determine the serum NO concentration, 10 mL fasting blood samples were taken. After sampling, the serum was separated using a centrifuge tool and was kept in −70 degrees centigrade. Enzyme-linked immunosorbent assay (ELISA) technique was used for determination of serum NO concentration.

### 2.5. Flow-Mediated Dilation Analysis

To assess the endothelial function, flow-mediated dilation (FMD) was evaluated. Forearm ischemia was induced by inflating a sphygmomanometer cuff to 50–100 mmHg for five minutes. Brachial artery diameter was assessed before ischemia (baseline brachial artery diameter) and immediately (<60 s) after deflation (brachial artery diameter after ischemia). Calculating the arterial diameter was done by ultrasonography method. The FMD% was calculated according to the following formula as a measure of brachial artery endothelial function; FMD% = [(maximum diameter − baseline diameter)/baseline diameter] × 100 [10, 11].

### 2.6. Data Analysis

Statistical analysis was performed using SPSS for Windows (Version 16.0, 2007, SPSS Inc., Chicago, IL, USA). Paired *t*-test and Student's *t*-test for continuous variables and chi-square test for categorical variables were used to compare variables. Statistical significance was assessed at the 0.05 probability level in all analyses. All the values are given as mean ± standard deviation (mean ± SD) or numbers (%).

## 3. Results

**Figure 1:**
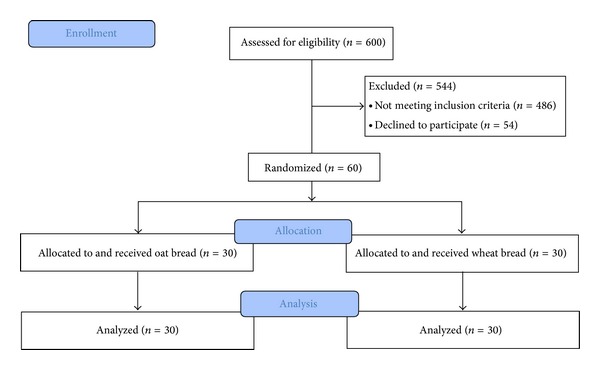
Patients flow diagram.

A total of 600 patients were contacted by phone and evaluated for being invited for the study. From 150 invited patients, 100 came to our center for further evaluations. From these patients, 64 were eligible to participate. Four patients discontinued before the intervention. Finally 60 patients started the experiment and completed the study (Figure 1). Mean age of the participants was 51.1 ± 9.3 years and 65% (*n* = 39) were female. There was no difference between the intervention and control groups in age (52.67 ± 8.90 versus 49.57 ± 9.60 years; *P* = 0.200), gender (female 63.3% versus 66.7%; *P* = 0.787), or other baseline characteristics (Table 1).

After the intervention, there was a significant reduction in weight in both groups, but reduction of BMI was significant only in the intervention group (*P* = 0.029). No significant change was observed in other anthropometric or cardiovascular measures after intervention (Table 1).

Comparison between the two study groups for NO and FMD is summarized in Table 2. There was no significant difference between the intervention and control groups in NO concentration before or after the study. Split analysis revealed that NO concentration increased significantly in the intervention (*P* = 0.017) but not in the control group (*P* = 0.530).

Analysis of brachial artery diameter showed that there were no significant differences between intervention and control group before and after study when comparing the baseline and after ischemia diameters. But, baseline brachial artery diameter and also diameters after ischemia had significant increase after the intervention in oat bread consumers (*P* = 0.005 and *P* < 0.001, respectively). In contrast, in the control group there were no significant changes in baseline and after ischemia artery diameters after the study. FMDs were significantly higher in the intervention group than the control group before and after the intervention. But, changes in FMDs were not different within or between the groups after intervention (Table 2).

## 4. Discussion

The aim of the current study was to assess the effects of bread containing oat bran on serum NO levels and endothelial dysfunction in patients with hypercholesterolemia. According to our results, oat bread consumption increases NO concentration. Although baseline brachial artery diameter and diameters after ischemia had significant increases after oat bread consumption, analyses showed no effect on FMD.

It is well known that dietary fibers have an important role in prevention and treatment of various diseases [5]. Queenan et al. found that oat beta-glucan consumption for six weeks in hypercholesterolemic adults can significantly lower total cholesterol and LDL levels [8]. Some other studies showed similar results in this regard [12–14]. Another study has revealed that reduction of serum cholesterol level and treatment of hyperlipidemia are associated with improvement of FMD [15]. Consumption of oat bread is also reported to be effective in control of diabetes mellitus and improvement of plasma glucose level [16, 17].

Endothelial dysfunction referred to different pathological conditions but, in most of the literatures, it means impaired endothelium dependent vasorelaxation caused by loss of NO in the vessels [18]. Inactivation of NO will occur in many diseases such as hypertension, hypercholesterolemia, and diabetes [18, 19]. According to the survey of Williams et al., NO mediated vasodilation is impaired in patients with diabetes mellitus [20]. Our results showed that oat bread consumption for 4 weeks significantly increases the serum NO level and brachial artery diameters (both before and after ischemia). However, we found no effect on FMD. The increase in brachial artery diameters after oat bread consumption might be due to the increase in NO production. Accordingly, the response of a predilated vessel to ischemia is less evident by assessing the FMD. Indeed, the FMD may be confounded by the baseline brachial artery diameter; a large increase in baseline brachial artery diameter may be accompanied by a decrease in FMD as a result of the change in resting tone, not a real change in endothelial function [21]. Applying a NO synthase inhibitor such as L-NMMA and evaluation of L-NMMA-inhibitable FMD of the brachial artery after oat bread consumption would probably show the exact role of NO in this regard. Anyway, NO itself can be considered as an indicator of endothelial function and based on our study results it seems that consuming oat bread instead of wheat bread in hypercholesterolemic patients may prevent endothelial dysfunction and subsequent cardiovascular events, though these findings need more surveys to be confirmed.

Nutritionists recommend the intake of 35 grams per day dietary fibers for each person. Development and consumption of fiber-enriched foods will help achieve that goal [5]. Previous studies have mentioned various benefits for consumption of fibers. Lower BMI and preventing the obesity have been reported to be associated with higher intake of dietary fibers by consuming fruits and vegetables [22, 23]. Our results showed that significant decrease of BMI occurred in the oat bread consumers after the study period but not in the control group. Accordingly, replacing wheat bread with oat bread in hypercholesterolemic patients may prevent cardiovascular events not only through improving endothelial function but also by lowering the BMI.

Our study has some limitations. The randomization was not appropriate as the two groups were different in their baseline FMD, probably due to small sample size. We did not consider the previous usual diet of the participants, though patients underwent the same 2-week run-in period before intervention. So similar studies considering different risk factors are recommended. Also, evaluation of oxidative stress parameters which may be affected by beta-glucan and thus have effects on endothelial function would provide more information on the beneficial effects of oat bread consumption.

## 5. Conclusions

Consumption of oat bread for four weeks, compared with wheat bread, increases serum NO level and brachial artery diameters but has no effects on FMD. These findings reveal the importance of fiber consumption and suggest more strategies to improve dietary fibers especially in hypercholesterolemic patients. Further studies are warranted in this regard.

## Figures and Tables

**Table 1:** Comparison of cardiovascular characteristics between the two groups before and after the intervention.

	Groups		*P* value∗
	Oat bread *n* = 30	Wheat bread *n* = 30	
Weight (Kg)			
Before	77.2 ± 12.4	77.0 ± 14.0	0.965
After	76.2 ± 12.4	76.6 ± 13.9	0.911
*P* value^†^	0.020	0.037	
BMI (Kg/m^2^)			
Before	28.9 ± 3.5	28.9 ± 4.9	0.969
After	28.6 ± 3.5	28.8 ± 4.9	0.902
*P* value^†^	0.029	0.078	
Pulse rate (pulse/min)			
Before	71.3 ± 6.3	72.7 ± 5.9	0.402
After	71.7 ± 7.0	73.4 ± 6.4	0.334
*P* value^†^	0.803	0.487	
Systolic/diastolic BP (mmHg)			
Before	114.8 ± 10.9/77.0 ± 9.1	115.1 ± 14.6/76.3 ± 10.7	0.921/0.797
After	112.5 ± 12.1/76.3 ± 8.9	114.8 ± 13.5/75.3 ± 9.3	0.485/0.673
*P* value^†^	0.174/0.625	0.896/0.495	
Hip/waist circumference (cm)			
Before	105.3 ± 6.4/93.4 ± 9.9	106.6 ± 10.3/97.6 ± 11.6	0.546/0.141
After	104.9 ± 6.3/93.2 ± 9.5	105.9 ± 10.2/97.4 ± 11.9	0.626/0.133
*P* value^†^	0.231/0.623	0.033/0.824	

*Independent *t*-test, ^†^paired *t*-test, BMI: body mass index, and BP: blood pressure.

**Table 2:** Comparison of nitric oxide concentration and flow-mediated dilation between the intervention and control groups.

	Groups		*P* value∗
	Oat bread *n* = 30	Wheat bread *n* = 30	
Nitric oxide (*μ*mol/lit)			
Before intervention	263.2 ± 49.8	232.7 ± 57.2	0.689
After intervention	313.4 ± 62.9	250.2 ± 56.7	0.460
*P* value^†^	0.017	0.530	
NO changes (*μ*mol/lit)	50.2 ± 19.8	17.5 ± 27.5	0.337
Baseline brachial artery diameter (mm)			
Before intervention	3.48 ± 0.68	3.67 ± 0.54	0.252
After intervention	3.55 ± 0.67	3.66 ± 0.55	0.492
*P* value^†^	0.005	0.895	
Brachial artery diameter after ischemia (mm)			
Before intervention	4.15 ± 0.65	4.23 ± 0.54	0.607
After intervention	4.25 ± 0.66	4.24 ± 0.55	0.949
*P* value^†^	0.000	0.614	
Flow-mediated dilation (%)			
Before intervention	20.1 ± 7.3	15.8 ± 5.1	0.011
After intervention	20.6 ± 7.9	16.4 ± 7.0	0.034
*P* value^†^	0.546	0.533	
FMD changes (%)	0.48 ± 0.78	0.59 ± 0.92	0.932

*Independent *t*-test, ^†^paired *t*-test, NO: nitric oxide, and FMD: flow-mediated dilation.
